# Payload diversification: a key step in the development of antibody–drug conjugates

**DOI:** 10.1186/s13045-022-01397-y

**Published:** 2023-01-17

**Authors:** Louise Conilh, Lenka Sadilkova, Warren Viricel, Charles Dumontet

**Affiliations:** 1 Cancer Research Center of Lyon, UMR INSERM 1052; CNRS 5286, University of Lyon, Lyon, France; 2 Mablink Bioscience, Lyon, France; 3 Hospices Civils de Lyon, Lyon, France

**Keywords:** Antibody–drug conjugates, Payload, Topoisomerase 1 inhibitor, Cytotoxic molecule

## Abstract

Antibody–drug conjugates (ADCs) is a fast moving class of targeted biotherapeutics that currently combines the selectivity of monoclonal antibodies with the potency of a payload consisting of cytotoxic agents. For many years microtubule targeting and DNA-intercalating agents were at the forefront of ADC development. The recent approval and clinical success of trastuzumab deruxtecan (Enhertu^®^) and sacituzumab govitecan (Trodelvy^®^), two topoisomerase 1 inhibitor-based ADCs, has shown the potential of conjugating unconventional payloads with differentiated mechanisms of action. Among future developments in the ADC field, payload diversification is expected to play a key role as illustrated by a growing number of preclinical and clinical stage unconventional payload-conjugated ADCs. This review presents a comprehensive overview of validated, forgotten and newly developed payloads with different mechanisms of action.

## Statement of significance

The ability to cure cancer depends on the diversification of the mechanisms of action of the therapeutic compounds used. A number of compounds have been abandoned due to excessive toxicity. The conjugation of such agents with antibodies might provide a better therapeutic index and our presentation may therefore be of interest for a broad scientific community.

## Introduction

The use of monoclonal antibodies as therapeutic vector was hypothesized by Paul Ehrlich within the concept of the “Magic Bullet” [1]. Remarkable improvements in biology and chemistry led to the development of antibody–drug conjugates (ADCs), a new generation of biotherapeutics that combines the high specificity of antibodies to the potency of cytotoxic small molecules, with the aim to deliver highly potent payloads within the targeted cell. These weaponized antibodies could in certain cases significantly improve the therapeutic index of cytotoxic molecules and reduce their off-target toxicity, a major issue of conventional cytotoxic chemotherapies. Anticancer ADCs are comprised of three parts, a monoclonal antibody (mAb) which specifically recognizes an antigen on the target cell, a potent cytotoxic small molecule that triggers cell death when released, and a linker that binds these two elements together [2].

The complexity of these agents explains their arduous development, best exemplified by the chaotic history of gemtuzumab ozogamicin, which was first approved in 2000, removed from most markets in 2010 and then reapproved by the FDA in 2017 [3, 4]. Major improvements in mAb design, target selection, conjugation technologies, payload selection and standardization of quality controls have allowed this family to evolve into a mature component of the anticancer pharmacopeia with 13 agents currently approved for the treatment of cancer, among which seven have been approved during these past three years [5, 6].

Given the potency of the payload, the antibody must be highly selective for its target and maintain its half-life and biological properties after conjugation. Antibody engineering, such as Fc (fragment crystallizable) silencing or Fc capacity enhancement, is a potent tool that is developed to, respectively, balance antibody off-target toxicity induced by T-cell targeting or increase antibody functions such as ADCC and ADCP, and both methods have proven their clinical benefits in the context of therapeutic mAbs [2, 7, 8]. In the context of ADCs, it has been hypothesized that Fc silencing would considerably reduce off-target toxicity. Thrombocytopenia and neutropenia are common adverse effects observed in patients treated with ADCs that could relate to the expression of FcγRIIa receptor at the surface of platelets [9]. MEDI4276, an analogue of trastuzumab emtansine (T-DM1, Kadcyla^®^) with reduced FcγR binding, has been explored in clinical trials, aiming to reduce thrombocytopenia observed with T-DM1 [10] (NCT02576548). Surprisingly this ADC has demonstrated significant toxicity in a first-in-human trial [11]. In contrast, Fc capacity enhancement has proven its benefits with the approval of brentuximab vedotin (Blenrep^®^), whose mAb is afucosylated. The recent development of mAb derivatives widens vector possibilities while aiming to improve essential characteristics of ADC carriers. Smaller formats such as mAb fragments (scFv, single-chain variable fragment, and Fabs, fragment antigen-binding) have been explored in this context (small-format drug conjugates) with the aim to enhance solid tumor penetration and cell internalization [12, 13]. However, to date, no such candidates have entered clinical trials as they were found to face rapid elimination and may consequently not present benefits over classical mAb formats [14]. In contrast, another family of small-format drug conjugates based on bicycle peptides seems to meet the challenge of competing with mAb format conjugates with competitive uptake efficiency, as illustrated with the three bicycle peptide conjugates currently in clinical trials (NCT04561362, NCT04180371 and NCT03486730). Other important parameters than the size impact on pharmacokinetics (PK) and circulating half-life of these macromolecules, including chemical modifications, affecting their retention by the kidney tubular epithelium, as well as their recycling rate through the neonatal Fc receptor (FcRn) [15, 16]. Small-format conjugates may benefit from the modulation of these parameters. Multivalent binding entities such as diabodies or bispecifics are being developed to improve antigen affinity, selectivity, or internalization and could constitute promising vectors [17].

The most widely applied bioconjugation methods use lysine side-chain amines and cysteine interchain thiols [18]. However, the heterogeneity of the resulting mixture has been a major issue in ADC failure. Other conjugation strategies have been developed, including site-specific conjugation through specific or engineered amino acids, but failed to demonstrate improved outcome in clinical trials at this time. Iladatuzumab vedotin (DCDS0780A), a THIOMAB™ version of the FDA-approved polatuzumab vedotin (Polivy^®^), was explored in clinical trials but failed to reach phase II due to excessive ocular toxicity at the tested doses [19]. This THIOMAB™-drug conjugate’s technology demonstrated a great potential, but the difference in the two mAbs and/or in the clinical design, including the selected doses, indication and patient population may have resulted in the approval of one and not the other. It is important to highlight in this context that it is not only the technology that matters. Novel conjugation strategies such as glycans and short peptide tags (enzyme-assisted ligation) or more recently through ADP-ribosyl cyclase are being explored with the aim to generate homogeneous and physically stable ADCs [20–25].

The linker plays a major role in ADC design since it strongly impacts on the safety, potency and activity of the ADC. Most importantly, the linker is expected to remain stable in circulation to avoid premature detachment of the drug while allowing its release within the targeted cell. Two categories of linkers have been developed and can be distinguished based on their cleavability. Cleavable linkers are either sensitive to pH for hydrazone linkers, to glutathione or disulfide isomerase for disulfide linkers and to proteases such as cathepsin B for dipeptide bonds. Non-cleavable linkers rely on lysosomal degradation of the antibody moiety, thereby conserving at least one amino acid, most commonly lysine or cysteine, attached to the payload-linker complex. This approach improves the linkage stability since antibody digestion is required for payload release. While it is emphasized that the more stable the linker is, the less off-target toxicity it triggers, these technologies were often found to be too stringent to support anti-tumoral activity. Safety of ADCs remains a major challenge in their design and on-target in addition to off-target toxicity is not only driven by the instability of the linker-payload. On-target toxicity is rather always driven by the mAb and its affinity/avidity to the target, together with the payloads’ mechanism of action, again illustrating how important the match between tumor type, target antigen and ADC construction is recent interest in linker design improvement has led to the development of hydrophilic linkers to balance payload hydrophobicity [26–29]. Sulfonate, polyethylene glycol (PEG), polysarcosine (PSAR) or more recently DNA-based linkers have significantly improved ADC stability and pharmacokinetics, leading to less toxic and more active ADCs [30–37].

The drug-to-antibody ratio (DAR) has until recently been maintained under a value of four to avoid mAb aggregation and limit the overall hydrophobicity of ADCs, which has been reported to be correlated with toxicity, reduced half-life and a narrow therapeutic index. Increased DAR is therefore more suitable for less hydrophobic, or well-compensated hydrophobic payloads, as illustrated with novel linker technologies including masking entities, or linker hydrophilic inserts, whose efficiency directly determines the capacity of DAR increase [35, 38]. Restoration of hydrophilicity and naked-like mAb pharmacokinetic profile in addition to a high DAR considerably increase the payload’s exposure to the tumor. These new linker technologies have enabled the development of less potent payloads than DNA-intercalating or microtubule-disrupting agents, as illustrated with the approval of two topoisomerase 1-based ADCs conjugated at DAR8, trastuzumab deruxtecan (Enhertu^®^) and sacituzumab govitecan (Trodelvy^®^). These highly loaded ADCs with unconventional payloads could also potentially widen the therapeutic indications of ADCs by addressing tumors with high or low target expression levels, as illustrated with the recent approval of Enhertu^®^ in HER2-low-expressing tumors based on the results of DESTINY-Breast04. In contrast, in the context of highly potent payloads, a lower DAR has so far been preferable, as illustrated with the recent approval of loncastuximab tesirine (Zynlonta^®^), a DAR2 of extremely potent pyrrolobenzodiazepines (PBDs) payload. Of note, unlike trastuzumab deruxtecan and analogues evaluated in clinical trials that are conjugated at DAR8, datopotamab deruxtecan’s DAR is lowered to 4 to reduce the toxicity driven by its target [39], illustrating the multidimensional design of ADCs.

**Fig. 1:**
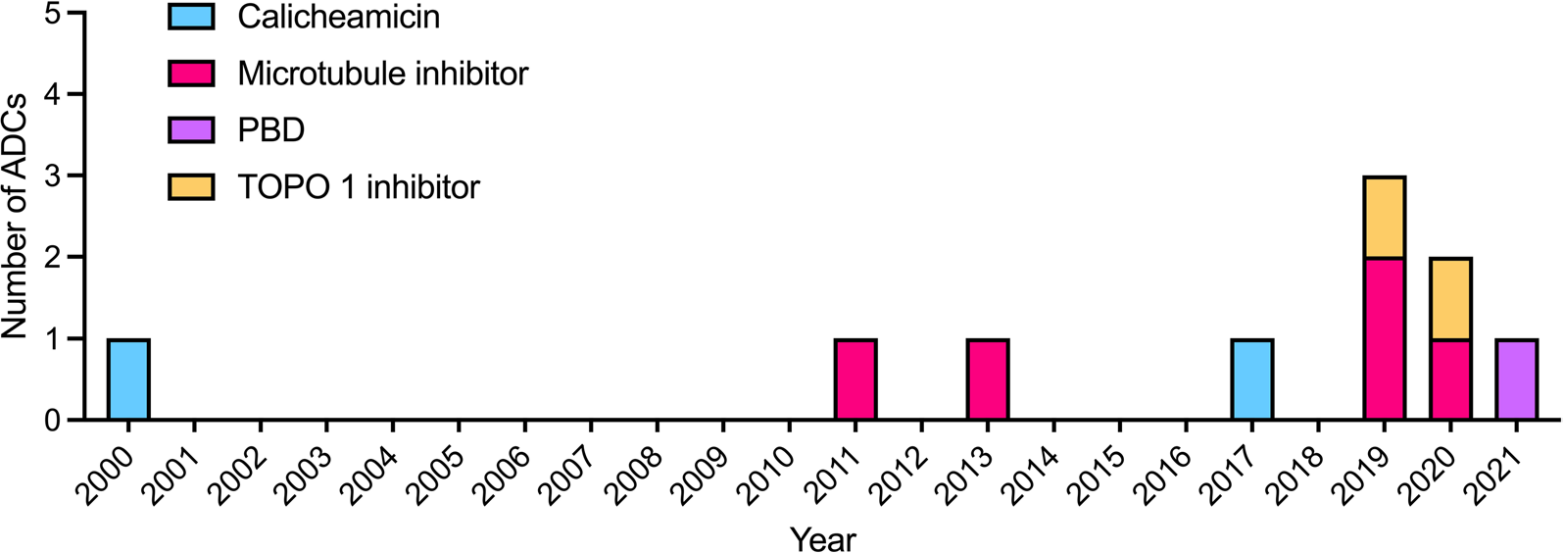
FDA approval of anticancer ADCs. ADCs are identified according to the nature of their payload: Microtubule-disrupting agents; DNA-targeting agents: Calicheamicin, pyrrolobenzodiazepine (PBD), topoisomerase 1 (TOPO 1) inhibitor

The payload (also designated as warhead) exerts the ADC’s intracellular cytotoxic activity. The nature of the cytotoxic agent covalently bound to the antibody through the linker moiety is of great importance since its mechanisms of action will determine the resulting ADC's potency as an anticancer compound and its possible indications. First-generation ADCs, coupled to conventional chemotherapeutics (taxoids, anthracyclines), lacked efficacy as the payloads were not potent enough since only a small fraction of the total conjugates administrered successfully delivered their payload within the target cell [2, 40]. Tumor penetration, target copy number at the cell surface and ADC internalization and degradation strongly impact the intracellular concentration of the free payload. The payload must therefore be highly potent at low concentration, with 50% inhibitory concentrations (IC_50_s) in the low to sub-nanomolar range [41]. Other factors such as molecule stability in plasma and under acidic conditions, accessibility of a conjugation site or solubility are crucial [42]. For many years, payloads were essentially represented by 2 categories: microtubule inhibitors, including maytansinoids and auristatins and DNA-alkylating agents, such as calicheamicins. These payloads lead to the approval of eight ADCs (Fig. 1).

Newer and more potent DNA-alkylating agents such as PBD monomers and dimers, indolino-benzodiazepines (IGNs) or cyclopropabenzindolone (CBI) monomers and dimers, with IC_50_ values in the picomolar range, have been at the forefront of ADC design [43, 44]. These molecules are among the most potent anti-tumor chemicals ever synthesized, and their specific targeting to tumor cells through an ADC construction was investigated to generate highly potent “magic bullets.” However, strong dose-limiting toxicities have limited their clinical development [45, 46] and currently only loncastuximab tesirine has obtained FDA approval in 2021 (Fig. 1) [47].

Efforts have been made to diversify payload families to molecules with original mechanisms of action, including several which do not directly target DNA or microtubules. Less potent molecules have benefited from major breakthroughs in ADC construction, through improved linker design allowing higher DAR values, more stable payload attachment or enhanced bystander killing activity. A recent and spectacular success has been the development of topoisomerase 1 (topo-1) inhibitors, which represented a turning point in payload selection, with the approval of two topo-1 inhibitor-based ADCs since 2019 (Fig. 1), trastuzumab deruxtecan (Enhertu^®^, DS-8201a) and sacituzumab govitecan (Trodelvy^®^) [48, 49]. Recent reviews have described the landscape of validated and exploratory therapeutic targets for ADCs [50, 51]. This review aims to describe the landscape of validated, forgotten and newly developed payloads in ADC context with diverse mechanisms of action, excluding microtubule inhibitors and DNA-alkylating agents.

## A successful payload family: Topoisomerase 1 inhibitors

**Fig. 2:**
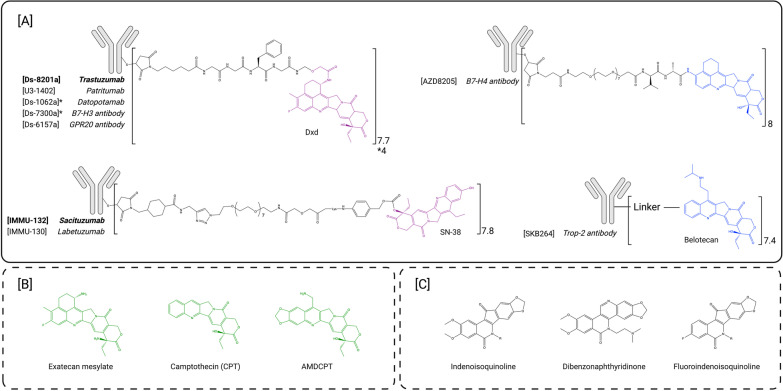
Structure of topoisomerase I inhibitors-based ADCs. **A** FDA-approved ADCs and payloads (purple) and ADCs and payloads under clinical evaluation (blue). **B** Topoisomerase 1 inhibitors used in preclinical development (green). **C** Next-generation topoisomerase I inhibitors as potential payloads for ADCs. Notations within the figure: [ADC name], antibody, payload

Topoisomerase 1 inhibitors constitute the most recent antibody–drug conjugate payload family to be approved by the FDA, first driven by trastuzumab deruxtecan, followed by sacituzumab govitecan (Table 1, Fig. 2A) [48, 49]. The recent development of these conjugates based on moderately potent payloads has been enabled by the production of highly loaded ADCs with DAR values of 8 [52, 53].

**Table 1:** Landscape of topoisomerase 1 inhibitors antibody–drug conjugates

Payload name	ADC name	Antibody	Antibody target	linker	Conjugation site	DAR	Development status	Indication	Last publication date or clinical trial status	Reference
Dxd	**DsS8201a, trastuzumab deruxtecan, Enhertu**	**trastuzumab**	**HER2**	**mc-GGFG-AM cleavable**	**cysteine**	**8**	**FDA approved**	**unresectable or metastatic HER2 + BC, gastric or gastroesophageal carcinoma**	**N/A**	**48**
	*U3-1402, patritumab deruxtecan*	*patritumab*	*HER3*	*mc-GGFG-AM cleavable*	*cysteine*	*8*	*phase II*	*NSCLC, metastatic colorectal, metastatic BC and BC*	*Recruiting or active*	*NCT04479436, NCT03260491, NCT02980341, NCT04619004, NCT04676477, NCT04699630, NCT04610528, NCT0496576*
	*Ds-1062a, datopotamab deruxtecan*	*datopotamab*	*TROP2*	*mc-GGFG-AM cleavable*	*cysteine*	*4*	*phase III*	*NSCLC, metastatic lung cancer, TNBC, HR* + *BC, BC*	*Recruiting or active*	*NCT02923115, NCT03742102, NCT04526691, NCT04612751, NCT05104866, NCT03401385, NCT04940325, NCT04656652, NCT04484142, NCT05215340*
	*Ds-7300a*	*B7-H3 antibody*	*B7-H3*	*mc-GGFG-AM cleavable*	*cysteine*	*4*	*phase I/II*	*Advanced solid malignant tumors*	*Recruiting or active*	*NCT04145622*
	*Ds-6157a*	*GPR20 antibody*	*GPR20*	*mc-GGFG-AM cleavable*	*cysteine*	*8*	*Phase I*	*GIST*	*Recruiting or active*	*NCT04276415*
SN-38	**IMMU-132, sacituzumab govitecan, Trodelvy TM**	**sacituzumab (hRS7)**	**TROP2**	**Cleavable CL2A (hydrolyzable)**	**cysteine**	**7.6**	**FDA approved**	**HER + /HER-Metastatic BC (mTNBC)**	**N/A**	**49**
	*IMMU-130*	*labetuzumab (hMN-14)*	*CEACAM5*	*Cleavable CL2A (pH-sensitive)*	*cysteine*	*7.6*	*Phase II (withdrawn)*	*Solid tumors (metastatic colorectal; colon cancer; rectal cancer)*	*2020 (Phase II withdrawal)*	*NCT01270698, NCT01605318, NCT01915472*
	***IMMU-140***	***IMMU–112 (hL243)***	***HLA-DR***	***Cleavable CL2A (pH-sensitive)***	***cysteine***	***6.1***	***late preclinical***	***ALL, CLL, MM, AML, DLBCL, HL and melanoma***	***2018***	***87***
		***Epratuzumab (hLL2)***	***CD22***	***Cleavable CL2A (pH-sensitive) or CL2E (cathepsin cleavable)***	***cysteine***	***6***	***preclinical***	***B cell malignancy, Lymphoma and leukemia***	***2012***	***88***
		***Veltuzumab***	***CD20***	***Cleavable CL2A (pH-sensitive) or CL2E (cathepsin cleavable)***	***cysteine***	***6***	***preclinical***	***B cell malignancy***	***2012***	***88***
		***Milatuzumab***	***CD74***	***Cleavable CL2A (pH-sensitive) or CL2E (cathepsin cleavable)***	***cysteine***	***6.5–6.6***	***Preclinical***	***solid cancers (and lymphoma)***	***2013***	***89***
		***Trastuzumab***	***HER2***	***ester bond or carbonate cleavable (± PEG4)***	***Cysteine***	***3.2–3.7***	***preclinical***	***ovarian***	***2015***	***90***
		***A7R***	***IL-7R***	***Carbamate bond + PEG12***	***Cysteine***	***4 to 6***	***late preclinical***	***IL-7R + Lymphoid malignancies, autoimmune diseases, metastatic solid tumors***	***2018***	***93***
		***rituximab***	***CD20***	***Carbamate or ester cleavable + PEG27***	***cysteine***	***7***	***preclinical***	***Lymphoma***	***2013***	***91***
		***B8-4***	***EpCam***	***Carbamate or ester cleavable + PEG27***	***cysteine***	***8.5***	***preclinical***	***Pancreatic cancer***	***2013***	***91***
		***35–4***	***Collagen-4***	***Carbamate or ester cleavable + PEG27***	***cysteine***	***7.5***	***preclinical***	***hypovascular stroma-rich tumor (pancreatic)***	***2013***	***91***
AZ'0132 (exatecan derivative)	*AZD8205*	*B7-H4 antibody*	*n.d*	*n.d*	*n.d*	*8*	*Phase I/Phase II*	*breast, ovarian and endometrial cancers and cholangiocarcinoma*	*2021*	*NCT05123482, 69*
KL610023 (belotecan derivative)	*SKB-264*	*n.d*	*TROP2*	*n.d*	*n.d*	*7.4*	*Phase I/II*	*ovarian epithelial and breast cancer, gastric and urothelial carcinoma, NSCLC, SCLC*	*Recruiting or active*	*NCT04152499*
Exatecan mesylate		***Trastuzumab***	***HER2***	***cleavable glucuronide + PSARLink***	***cysteine***	***8***	***preclinical***	***HER2 + solid tumors***	***2021***	***36***
	***PRO1184***	***Fra antibody***	***Fra***	***hydrophilic protease cleavable***	***cysteine***	***8***	***late preclinical***	***NSCLC, ovarian***	***2022***	***77***
	***PRO1102***	***Trastuzumab***	***HER2***	***hydrophilic protease cleavable***	***cysteine***	***8***	***POC***		***2022***	***76***
	***PRO1160***	***CD70 antibody***	***CD70***	***hydrophilic protease cleavable***	***cysteine***	***8***	***preclinical***		***2022***	***78***
7-n-butyl-10-amino-CPT		***cAC10***	***CD30***	***mc-Val-cit-PAB or glucuronide cleavable linkers***	***cysteine***	***4 to 8***	***preclinical***	***hematologic malignancies***	***2009***	***94***
		***h1F6***	***CD70***	***mc-Val-cit-PAB or glucuronide cleavable linkers***	***cysteine***	***4 to 8***	***preclinical***	***hematologic malignancies and renal cell carcinoma***	***2009***	***94***
		***cBR96***	***LeY***	***mc-Val-cit-PAB or glucuronide cleavable linkers***	***cysteine***	***4 to 8***	***preclinical***	***carcinoma***	***2009***	***94***
7-n-butyl-9-amino-10, 11-MDO-CPT		***cAC10***	***CD30***	***mc-Val-cit-PAB or glucuronide cleavable linkers***	***cysteine***	***4 to 8***	***preclinical***	***hematologic malignancies***	***2009***	***94***
		***h1F6***	***CD70***	***mc-Val-cit-PAB or glucuronide cleavable linkers***	***cysteine***	***4 to 8***	***preclinical***	***hematologic malignancies and renal cell carcinoma***	***2009***	***94***
		***cBR96***	***LeY***	***mc-Val-cit-PAB or glucuronide cleavable linkers***	***cysteine***	***4 to 8***	***preclinical***	***carcinoma***	***2009***	***94***
CPT		***trastuzumab***	***HER2***	***Pt–PEG, ester cleavage***	***cysteine***	***2.5–4***	***POC***	***Solid tumors***	***2017***	***95***
		***cetuximab***	***EGFR***	***Pt–PEG, ester cleavage***	***cysteine***	***2.5–4***	***POC***	***Solid tumors***	***2017***	***95***
		***rituximab***	***CD20***	***Pt–PEG, ester cleavage***	***cysteine***	***2.5–4***	***POC***	***N/A***	***2017***	***95***
AMDCPT	***SGN-CD30c***	***cAC10***	***CD30***	***maleimidopropionyl-PEG7-valine-lysine-glycine***	***cysteine***	***8***	***late preclinical***	***Relapse and refractory lymphoma***	***2020***	***97, 98***
		***cAC10***	***CD30***	***val-lysine-glycine tripeptide ± PEG4 or PEG8***	***cysteine***	***8***	***preclinical***	***lymphoma***	***2021***	***96***
other CPT derivative			***EGFR or Fra***	***mc-AAA-AM cleavable ± polyhydroxyl moiety***	***cysteine***	***6.4–7.5***	***preclinical***	***solid tumors***	***2019***	***79***

FDA-approved (Bold), clinically evaluated (Italic) and preclinically developed ADCs are described. *ADC* Antibody–drug conjugate, *DAR* drug-to-antibody ratio, *FDA* Food and Drug Administration

Topoisomerase enzymes are located within the cell nucleus. Their role is to control and repair DNA supercoiling and entanglements occurring during DNA opening, upstream transcription and replication. These catalytic enzymes cleave, repair supercoils and re-ligate DNA strands. Topoisomerases are divided into two families differentiated by their cleavage activity: topoisomerases I cleave single-stranded DNA while topoisomerases II cleave double-stranded DNA. Topoisomerase inhibitors specifically bind to the interface of DNA-topoisomerase complexes, thereby inhibiting the topoisomerase repair machinery and leading to DNA damage and consequently cell apoptosis [54–56]. The most potent topoisomerase inhibitors, however, are 100 to 1000-fold less potent than maytansines or calicheamicin, explaining the initial lack of interest for this payload class in initial ADC design [57].

This payload class includes camptothecin- and non-camptothecin-based compounds. Camptothecin (CPT) is a natural plant alkaloid composed of five chemical rings which is poorly water-soluble (Fig. 2B). Several derivatives that present improved bioavailability have been approved by regulatory authorities, namely topotecan, irinotecan and belotecan [58, 59]. These agents have been approved in several indications including ovarian, lung, cervical and colon cancers. A liposomal formulation of irinotecan has also been approved for the treatment of advanced pancreatic cancer. Several other CPT derivatives have been synthetized such as gimatecan which is currently in phase II evaluation for the treatment of ovarian, fallopian tube or peritoneal cancers (NCT04846842). The most significant severe adverse events (SAEs) of CPT-based molecules include severe watery diarrhea, neutropenia and thrombocytopenia [60].

CPT derivatives have recently been used as ADC payloads due to their intermediate cytotoxic potency, with IC_50_ values in the low nanomolar range. Their potency lies in between those of very potent (picomolar IC_50_s) anti-microtubule/DNA-targeting agents and those of the conventional (micromolar IC_50_s) chemotherapy agents that were initially used in the very first ADC programs and failed for lack of efficacy reasons (methotrexate and doxorubicin). To date, two CPT derivatives have been successfully conjugated to antibodies and approved: DXd, and the active metabolite of irinotecan, SN-38 (Table 1).

### Exatecan and derivatives

DXd is a derivative of exatecan (also known as DX8951f), a compound with increased activity and improved solubility compared to CPT, and described not to be an ABCC2 or ABCG1 substrate [61]. Unconjugated exatecan was evaluated in several clinical trials but its poor therapeutic window, with dose-limiting neutropenia and thrombocytopenia and strong gastrointestinal toxicity, did not allow an improvement in survival rates [62]. In a first attempt, bioconjugation of exatecan onto antibodies led to partial success with significant aggregation of the conjugate. This issue was solved by Daiichi Sankyo scientists by using a slightly modified glycolic acid derivative of exatecan, named DXd. It was found that this new compound retained exatecan potency, while enabling the successful bioconjugation of up to 8 DXd molecules per antibody without significant aggregation. This deruxtecan drug-linker was used in several proprietary ADC programs, such as DS-8201a (Enhertu^®^), U3-1402 and DS-6157a, conjugated at DAR8 and DS-1062a and DS-7300a conjugated at lower DAR (4) to limit their toxicity [39], that are either approved by the FDA (DS-8201a) or currently under clinical evaluation (Table 1, Fig. 2A). Although the DXd payload presented lower passive membrane permeability than exatecan mesylate, it was found to be less myelotoxic and was therefore also selected for its improved safety profile [63].

Trastuzumab deruxtecan (DS-8201a or Enhertu^®^) is composed of the already approved HER2-targeting antibody trastuzumab, attached to 8 DXd payloads through a maleimide-based mc-GGFG-am protease cleavable linker (Fig. 2A). This innovative DAR8 ADC demonstrated an improved preclinical therapeutic window compared to first-generation ADCs, thanks to its optimized linker and payload [52, 61, 64–67]. Following two large phase 3 studies (DESTINY-Breast03, NCT03529110, DESTINY-Gastric01, NCT03329690), trastuzumab deruxtecan has been clinically approved by the FDA in 2019 for the treatment of unresectable or metastatic HER2 + breast cancer (BC) and in 2021 for the treatment of advanced or metastatic HER2 + gastric or gastroesophageal carcinoma and later in 2022 for the treatment of unresectable or metastatic HER2 + non-small cell lung cancer (DESTINY-Lung02) [68–70]. Importantly trastuzumab deruxtecan demonstrated strong antitumor activity in breast cancer patients who relapsed after treatment with trastuzumab emtansine and displayed more potent activity than irinotecan in patients with gastric cancer and durable anticancer activity in non-small cell lung cancer (NSCLC) and colorectal cancers [71–73]. Another breakthrough in ADC approval is illustrated by its clinical evaluation in the DESTINY-Breast04 trial that lead to its approval in 2022 for the treatment of unresectable or metastatic HER2-low breast cancer [74]. Several other clinical trials are currently ongoing, including DESTINY-breast05 and DESTINY-breast09 that, respectively, evaluate Enhertu in patients with residual disease after neo-adjuvant therapy in HER2 + BC or versus current first-line standard of care regimen in HER2 + BC, again illustrating its success. Four other ADCs that contain this promising linker-payload are currently under clinical evaluation for the treatment of solid tumors, by targeting either HER3 in NSCLC, metastatic colorectal and breast cancers, TROP2 in NSCLC and triple negative breast cancer (TNBC), B7-H3 in advanced solid tumors or GPR20 in gastrointestinal stromal tumors (GIST) (Table 1).

More recently, exatecan (Fig. 2B) has been preclinically explored as a potential ADC payload thanks to the development of hydrophilic cleavable linker architectures that are able to circumvent the hydrophobic and pro-aggregation characteristics of the compound. This allowed the conjugation of exatecan at elevated DAR values without disturbing the ADCs’ pharmacokinetic properties [36, 75, 76] (Table 1). These ADCs demonstrated strong antitumor activity in tumor xenografts and displayed a stronger bystander killing effect compared to deruxtecan-based ADCs thanks to the improved passive cell permeability of exatecan compared to DXd [36]. Two ADCs are being developed using this drug-linker strategy: PRO1184 and PRO1160 containing a hydrophilic exatecan-based linker are conjugated at DAR8 to, respectively, anti-FRa and anti-CD70 antibodies and are expected to enter clinical trials in 2023 [77, 78]. Recent in vivo studies have also demonstrated that exatecan does not require the fluorine ring function to exert its anti-tumoral activity thus widening the functionalization possibilities of the molecule to generate linkable derivatives (Table 1). The most promising ADC developed using this strategy (mAbE-21a, derivative 11, DAR7.5) demonstrated remarkable anti-tumor activity with complete remissions at 0.25 mg/kg in an EGFR+ model [79].

A novel proprietary exatecan derivative, AZ’0132, was disclosed this year and is being investigated as the payload of the ADC AZD8205 targeting B7-H4 [80] (Table 1, Fig. 2A). AZD8205 is currently undergoing phase I/phase II investigation for the treatment of breast, ovarian and endometrial cancers as well as cholangiocarcinoma (NCT05123482).

### Irinotecan

Irinotecan has been approved by the FDA for the treatment of various solid tumors such as gastrointestinal malignancies, glioblastomas and cervical cancer and is a pro-drug of the topoisomerase 1 inhibitor SN-38 [59]. SN-38 is water insoluble and causes severe toxicity including strong myelosuppression and high-grade diarrhea [81, 82]. Irinotecan was therefore developed to improve bioavailability and to obtain an acceptable therapeutic index. IMMU-132 (Trodelvy^®^) is an anti-TROP2 antibody conjugated to a SN-38 based drug-linker (Table 1, Fig. 2A) [49]. This ADC has been approved by the FDA in 2020 for the treatment of triple negative metastatic breast cancer [83] and metastatic urothelial cancer and is currently in clinical trials for the treatment of HR+ /HER2-, prostate and endometrial cancers (NCT03725761 and NCT04251416). Other ADCs have been developed with this SN-38-based linker including IMMU-130 (labetuzumab govitecan) [84–86] and IMMU-140 [87] targeting, respectively, CEACAM5 and HLA-DR (Table 1). Labetuzumab govitecan demonstrated acceptable toxicity and activity in phase I (NCT01270698), however, the phase II evaluation has been terminated in 2020 for undeclared reasons (NCT01915472). IMMU-140 is directed against HLA-DR and has shown promising preclinical activity both in hematological malignancies and melanoma [87]. SN-38 payload is also under preclinical evaluation against various liquid tumors (Table 1). To the best of our knowledge, despite promising preclinical results, none of these ADCs have entered clinical trials and the last related publications are over seven years old [88–92]. More recently, an A7R-SN-38 ADC has been developed for the treatment of autoimmune diseases, to circumvent steroid resistance (Table 1) [93].

### Belotecan derivative

Another topoisomerase 1 inhibitor, KL610023, which is a derivative of the FDA-approved molecule belotecan is being investigated as an ADC payload. This topoisomerase I inhibitor was developed to generate an anti-TROP2 ADC (SKB-264), currently in a phase I/II clinical trial (NCT04152499) in patients with various solid tumors (Table 1, Fig. 2A).

### Other topoisomerase 1 inhibitors

One of the limitations of camptothecin-based derivatives as ADC payloads is the lack of a linkable chemical amine group within the molecule. Other CPT derivatives have been synthetized to insert a linkable function within the payload without altering its anti-tumor properties (Table 1) [79, 94–96]. Among these derivatives, preclinical studies of cAC10, an anti-CD30 antibody conjugated to 8 AMDCPT molecules, have shown very promising results (Fig. 2B) [97, 98]. Several non-camptothecin derivatives have recently been developed, including indenoisoquinolines [99, 100], dibenzonaphthyridinone [101, 102] and fluoroindenoisoquinolines [103] (Fig. 2C). These molecules were shown to present several advantages compared to CPT derivatives including higher cytotoxicity, improved stability or prolonged activity and are currently in early phase clinical trials as small molecules. LMP-517, conjugated to a fluoroindenoisoquinoline, is being investigated; however, to the best of our knowledge, no data have been disclosed yet [104].

## Payloads that have reached clinical trials: promises and failures

While topoisomerase 1 inhibitors have profoundly modified the ADC payload landscape, several other agents have been evaluated in clinical trials. Table 2 summarizes original payloads which have been evaluated in patients. The main categories include topoisomerase 2 inhibitors, RNA polymerase inhibitors, Bcl-xL inhibitors and immune stimulants. In addition, glucocorticoids are now emerging as ADC payloads for indications beyond oncology.

**Table 2:** Clinical landscape of unconventional ADC payloads

	ADC description				Last known clinical development (starting–ending date)						
Payload mechanism	Payload name	ADC name	Antibody (or vector) name	Target	Phase I	Phase I/II	Phase II	Phase III	Indication	Sponsor	NCT number
*Oncology*											
Topoisomerase II inhibitors	Doxorubicin	SGN-15 (BMS-182248)	BR-96	Lewis-Y			1999–2005 (DISCONTINUED)		Le-Y expressing epithelial tumors, Hormone refractory prostate carcinoma, mBC, NSCLC	Seattle Genetics	NCT00086333, NCT00051571, NCT00031187, NCT00051584, NCT00028483
		IMMU-110 (hLL1-DOX)	Milatuzumab	CD74		July 2010–July 2013			multiple myeloma	Immunomedics (Gilead Science)	NCT01101594
	PNU-159682	NBE-002		ROR1		June 2020			TNBC, advanced solid tumor, advanced cancer	NBE therapeutics (Boehringer Ingelheim)	NCT04441099
		SOT102		CLDN18.2		April 2022			Gastric and pancreatic cancer	SOTIO	NCT05525286
RNApolII inhibitors	alpha-amanitin	HDP-101	J22.9-ISY	BCMA (CD-269)		May 2021			Multiple myeloma	Heidelberg Pharma	NCT04879043
Bcl-xL inhibitors	Clezutoclax	ABBV-155 (mirzotamab clezutoclax)	mirzotamab	B7-H3 (CD276)	July 2018				Advanced solid tumors	AbbVie	NCT03595059
DHFR inhibitors	Methotrexate	N/A	KS1/4		1990				NSCLC		105
TK inhibitors	Genistein	B43 genistein	B43	CD19	March 2000 (DISCONTINUED)				ALL and NHL	Parker Hughes Cancer Center	NCT00004858
Immune stimulants	TLR7/8 agonist	NJH395	Unknown	HER2 (ErbB2)	December 2018–2021 (COMPLETED)				Non-Brest HER2 + advanced malignancies	Novartis Pharmaceuticals	NCT03696771
		BDC-1001	Trastuzumab (biosimilar)	HER2 (ErbB2)		February 2020			Advanced and HER2-expressing solid tumors	Bolt Biotherapeutics	NCT04278144
	TLR8 agonist (zuvotolimod)	SBT6050	Pertuzumab	HER2 (ErbB2)		October 2021 (DISCONTINUED)			HER2-positive tumors	Silverback Therapeutics	NCT05091528, NCT04460456
	STING agonist (TAK-676)	TAK-500	CCR2 antibody	CCR2	October 2021				advanced or metastatic solid tumors	Takeda	NCT05070247, 188
*Beyond oncology*											
Glucocorticoids	Glucocorticoid receptor modulator	ABBV-3373	adalimumab (Humira^®^)	TNF-a			March 2019–Aug 2020 (COMPLETED)		Rheumatoid Arthritis	AbbVie	NCT03823391
		ABBV-154	n.d	TNF-a			May 2021		Rheumatoid Arthritis, Severely active Crohn's disease and Polymyalgia Rheumatica	AbbVie	NCT04888585, NCT05068284, NCT04972968
RNApolII inhibitors	dmDNA31	DSTA4637S (Anti-S. aureus TAC, RG-7861)	Human THIOMAB™ anti-S. aureus	Staphylococcus aureus	May 2017–Jan. 2020 (COMPLETED)				Bacteremia	Genentech, Inc	NCT03162250

*ADC* Antibody–drug conjugate, *DAR* drug-to-antibody ratio

### Topoisomerase 2 inhibitors

Topoisomerase 2 inhibitors are widely used in anticancer therapy in hematological malignancies and in solid tumors. Their mechanisms of action are complex and may involve not only direct inhibition of topoisomerase 2 activity but also DNA intercalation, ROS induction and mitochondrial disruption. Their toxicity profile includes myelosuppression, gastrointestinal toxicity and in some cases high-grade cardiotoxicity.

**Fig. 3:**
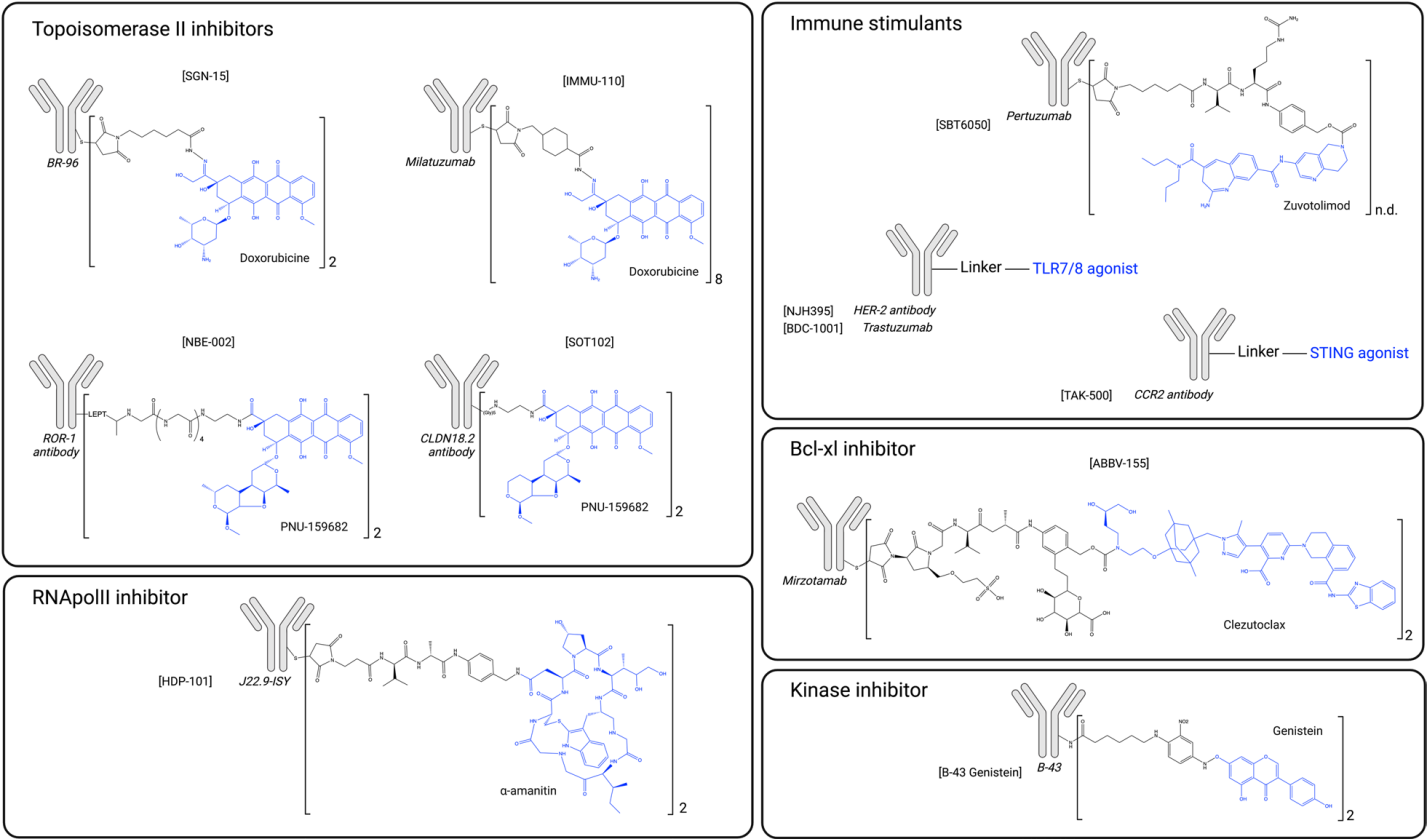
Structure of antibody–drug conjugates that have reach clinical trials and their payloads (blue) classified regarding their mechanism of action. Notations within the figure: [ADC name], *antibody,* payload

Doxorubicin has been used as first-line therapy for several decades for the treatment of breast, bladder and thyroid cancers, as well as lymphomas and multiple myeloma. Doxorubicin was also among the very first class of payloads used in ADC development, when conventional chemotherapeutic small molecules were first conjugated. The first ADC containing a topoisomerase 2 inhibitor (SGN-15, BMS-182248) was comprised of doxorubicin conjugated to the mouse BR-96 antibody, targeting Le-Y antigen (Table 2, Fig. 3). SGN-15 was developed at the very beginning of ADC discovery along with KS1/4-methotrexate (Table 2) [105], in the 1980s, for the treatment of prostate, breast and NSCL cancers. Its phase I clinical trial demonstrated acceptable tolerability, however the phase II led to off-target toxicities due to the instability of the linker and the expression of the Le-Y target in normal tissues. The ADC therefore lacked efficacy at the tolerated dose [106, 107]. Disappointing outcomes observed with the conjugation of already approved chemotherapies forged the consensus that an ADC payload should be much more potent than conventional chemotherapeutic agents. This led to the development of second-generation ADCs, conjugated to far more potent payloads, such as microtubule inhibitors and DNA-damaging agents [2].

In spite of these early unsatisfactory developments, doxorubicin was later conjugated to the CD74-targeting antibody milatuzumab (IMMU-110) for the treatment of multiple myeloma (Table 2, Fig. 3) [108]. This ADC was brought to clinical trials but demonstrated disappointing efficacy and its development was discontinued in 2013 (NCT01101594). Additionally, doxorubicin was used as an ADC payload in a preclinical linker proof of concept study (Table 3), having been conjugated either to a new cleavable linker (NEBI) [109] or a non-cleavable linker (SMAC) [110]. Results of the SMAC study showed that a non-cleavable linker may be too stringent for the development of a doxorubicin-based ADC, since no cytotoxicity was observed.

Given the lack of efficacy of doxorubicin as an ADC payload, another anthracycline, PNU-159682, which is 100-fold more cytotoxic than doxorubicin, was later explored. Besides being much more potent than other topoisomerase 2 inhibitors, PNU-159682 is not an efflux pump substrate. Payloads that are substrates for efflux pumps have been found to be limiting factors in ADC development [111]. In 2020, a novel PNU-159682-based ADC, NBE-002 (Table 2, Fig. 3), which targets ROR1, entered a phase I/II clinical trial (NCT04441099). Interestingly, NBE-002 induced long-term immune protection, which suggests that it could successfully be combined with immune checkpoint inhibitors (ICIs) [112]. SOT102 (formerly SO-N102) is another promising PNU-159682-based ADC that targets CLDN18.2 (Table 2, Fig. 3). SOT102 demonstrates a large therapeutic window in low-expressing tumors and has entered phase I clinical trials in April 2022 (EudraCT Number 2021–005,873-25) [113]. Many preclinical uses of PNU-159682 were also reported and results demonstrated its ability to by-pass mechanisms of resistance of usual payloads such as MMAE or DM1 (Table 3) [110, 111, 114–118]. The PNU-159682 payload was also conjugated alongside with MMAE to form a dual drug ADC (Table 3). However, while both mechanisms of action were simultaneously observed in vitro, no synergy was observed [119].

**Fig. 4:**
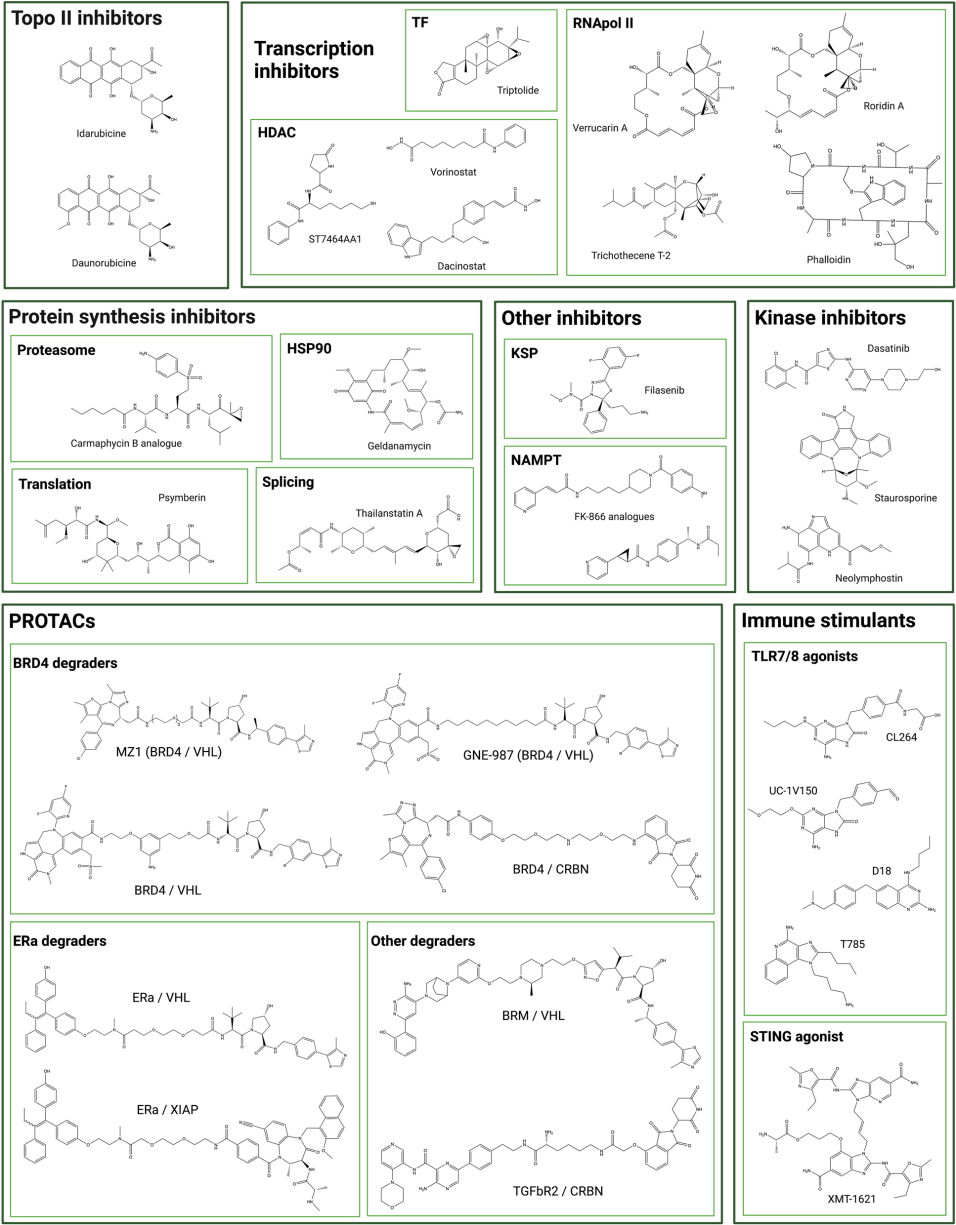
Chemical structure of unconventional ADC payloads conjugated at the preclinical stage

Daunorubicin and idarubicin conjugates were also developed at the preclinical stage in the 1990s (Table 3, Fig. 4) but displayed reduced efficacy [120–123]. An anti-HER2 affibody-idarubicin conjugate has more recently been evaluated in vitro with specificity to HER2-positive head and neck squamous cell carcinoma (HNSCC) cells rather than to HER2-positive BC cells (Table 3) [124].

### Transcription inhibitors

Transcription has a fundamental role in cell development, activity and proliferation and could therefore constitute an innovative and original target for an ADC payload. Transcription is regulated by RNA polymerase II (RNApolII) that directly binds to DNA and involves transcription factors that form complexes with RNApolII to initiate transcription (such as TFIIH) and co-regulators (such as histone deacetylases, HDAC) that mediate chromatin structure and accessibility. While some HDAC inhibitors have gained approval, there are currently no approved RNApolII inhibitors due to poor tolerability [125].

Amatoxins are natural and highly potent RNApolII inhibitors derived from the Amanita mushroom [126]. Alpha-amanitin and beta-amanitin together with seven other macrocyclic derivatives constitute the amatoxin family. Despite their extensive use as laboratory reagents to explore transcription mechanisms, alpha-amanitin proved to be far too toxic, particularly to the liver, to be further developed as an anticancer agent [127, 128]. However, this molecule presents numerous advantages as a potential ADC payload, including its original intracellular target, its favorable physicochemical properties (including hydrophilicity), its insensitivity to efflux pumps, and its ability to generate cytotoxicity in quiescent cancer cells [129]. By contrast amanitin’s hydrophilicity is expected to prevent neighbor cell killing via the bystander effect, which may restrict its use to homogeneously distributed targets. And even in those cases, complete lack of bystander effect may lead to a lack of efficacy as target distribution vary from a patient to another.

The amanitin derivative beta-amanitin was first conjugated in 1973 to albumin, and this ADC precursor demonstrated selective killing of macrophages (Table 3) [130]. This derivative was later conjugated to anti-MUC1 and anti-PSMA antibodies and demonstrated strong selective cytotoxicity in preclinical models (Table 3) [131–133]. Its analogue alpha-amanitin and its derivative azo-amanitin were also very early used as ADC payloads (Table 3) [134]. The azo-amanitin-ADC demonstrated approximately 500-fold higher cytotoxicity than the unconjugated molecule. This is explained by the hydrophilicity of the molecule that reduces cell membrane permeability, while it is efficiently internalized as an ADC construct. As of May 2021, the first amanitin-antibody conjugate (ATAC^®^) candidate, HDP-101, has entered an early phase clinical trial (Table 2, Fig. 3). HDP-101 is a BCMA-targeting ADC currently being evaluated in patients with multiple myeloma and plasma cell disorders (NCT04879043) [135]. ATACs were recently characterized as immune activating drugs. They were found to induce immunogenic cell death (ICD) and to exhibit synergy with ICI which opens new horizons for combination possibilities in the clinical setting [136]. Of note, a number of alpha-amanitin ADCs directed against other targets (EpCam, HER2, PSMA, CD19) have displayed potent anti-tumor activity both in vitro and in vivo (Table 3) [137, 138]. Alpha-amanitin has also been conjugated as a dual warhead alongside with MMAE (Table 3) [139]. This DAR 1 + 1 ADC targets FGFR1 and has demonstrated a potent in vitro cytotoxicity. Other highly potent RNApolII inhibitors were conjugated in the 1990s, such as phalloidin, and the mycotoxins trichothecene, verrucarin A and roridin A (Table 3, Fig. 4) [140, 141]. Considering how much ADC design has progressed since the 90s, and the nanomolar cytotoxicity of these compounds in various cell lines, these molecules may be the object of further exploration in the coming years [142].

Another strategy to stop DNA transcription is to inhibit transcription factors (TFs). TFs are essential to RNApolII attachment to the DNA at the initiation step [143]. TF inhibitors (TFi) have already demonstrated their anti-tumor activity in clinical trials with the water-soluble pro-drug minnelide, currently in phase II evaluation (NCT04896073). Triptolide, a natural compound derived from the Chinese medicinal herb called "thunder god vine", is highly cytotoxic but is also hydrophobic, presents poor bioavailability and high toxicity (Fig. 4). Efforts are therefore being made to develop analogues with better pharmacochemical properties [144]. Another strategy would be to conjugate this molecule to a targeting entity, thus by-passing these issues. Triptolide was recently conjugated for the first time to an anti-CD26 antibody to target mesotheliomas and lymphomas (Table 3) [145]. This non-cleavable ADC efficiently stopped mRNA synthesis in targeted cells and presented promising in vitro and in vivo antitumor activity. A cetuximab–triptolide ADC was also developed for the treatment of EGFR-positive lung cancers (Table 3) [146]. This ADC presented selectivity toward EGFR-overexpressing models and lower toxicity than unconjugated triptolide. Cetuximab–triptolide efficiently induced transcription inhibition, with potent in vitro and in vivo anti-tumor activity. A HER2-targeting triptolide ADC was also evaluated with similar results [147]. However, for each triptolide-based ADC, high doses were required to observe anti-tumor activity in xenograft models and no maximum tolerated dose was reported in these papers, thus questioning the width of the therapeutic index.

HDACs (histone deacetylases) impact on transcription factors and are therefore involved in various cellular processes including transcription. They have been found to be overexpressed or overactivated in cancer cells and are thought to be involved in increased proliferation, migration and invasion [148, 149]. Vorinostat and dacinostat are two examples of FDA-approved HDAC inhibitors (HDACi). These molecules however present strong risks of systemic side effects such as thrombocytopenia and gastrointestinal toxicity, and a poor PK profile. They have been studied in ADC design since 2018: ST74612AA1 is the first bioconjugated HDAC inhibitor (Table 3, Fig. 4). This relatively non-toxic molecule is a second-generation pan-HDACi. This molecule was conjugated to cetuximab and trastuzumab, and both ADCs presented a safer profile than unconjugated HDACi, while being active in cell-line-derived xenograft (CDX) and patient-derived xenograft (PDX) models [150, 151]. However, as was observed with TFi-based ADCs, xenograft models were treated with the high dosing of 30 mg/kg. In 2020, vorinostat and dacinostat were also conjugated to cetuximab and trastuzumab with interesting anti-proliferative activity in vitro (Table 3, Fig. 4) [152].

### Bcl-xL inhibitors

Bcl-2 family members can either be pro (Bad, Bim, PUMA, Bik, Bak, Bax, etc.)- or anti-apoptotic proteins (Bcl-2, Bcl-xL, Bcl-w, Mcl-1, etc.). In cancer cells, the equilibrium between these proteins is generally tilted toward survival, making anti-apoptotic proteins interesting and original targets for an innovative ADC payload [153].

Bcl-xL and Bcl-2 inhibitors are classified according to their chemical function scaffold into 4 major families: N-acylsulfonamides (navitoclax, venetoclax), indoles (obatoclax), gossypol acetic acid (AT-101, sabutoclax) and benzothiazole hydrazones (such as WEHI-539) [154–159]. Inhibition of Bcl-xL has been associated with profound thrombocytopenia, justifying the search for highly specific Bcl-2 inhibitors such as venetoclax [160]. Currently venetoclax is approved in a subgroup of patients with chronic lymphocytic leukemia and in acute myeloid leukemia [161].

ABBV-155 (mirzotamab clezutoclax) is an anti-B7-H3 antibody conjugated to the Bcl-xL inhibitor clezutoclax (Table 2, Fig. 3). This innovative ADC entered an ongoing phase I/II clinical trial in 2018 for the treatment of advanced solid tumors as a single agent and in combination with paclitaxel in patients with advanced non-small cell lung cancer and breast cancer (NCT03595059). No dose-limiting toxicities were reported in the first 31 patients included in the single agent phase 1 cohort, with SAEs consisting of anemia, decreased lymphocyte count, fatigue and diarrhea. Partial responses were observed in the paclitaxel combination arm in 21% of patients.

### Tyrosine kinase inhibitors

The human kinome comprises over 500 kinases, among which more than 150 are associated with various diseases including cancers. Protein kinases are enzymes that catalyze phosphorylation and are divided into 3 categories: serine, threonine or tyrosine kinases. Over a quarter of small molecules currently being investigated in clinical trials are protein kinase inhibitors and more than 30 FDA-approved molecules for cancer treatment are kinase inhibitors. In cancer a variety of kinase families are involved in cell cycle progression, cell proliferation, motility and angiogenesis. Since the approval of the first kinase inhibitor imatinib in 2001, kinase inhibitors have been classified into 5 categories: Types I and II are ATP competitive, respectively targeting the active or inactive form of the kinase; type III binds to an allosteric pocket of ATP; type IV to an allosteric pocket of the kinase, and type V combines multiple binding modes [162].

While being largely explored for cancer treatment, protein kinase inhibitors have not been extensively explored as ADC payloads presumably because of their low potency. The anti-CD19 antibody B43 has been conjugated to genistein, an isoflavone phytoestrogen contained in soybean, which was found to induce apoptosis and cell proliferation inhibition via the inhibition of epidermal growth factor receptor (EGFR), a tyrosine kinase receptor (Table 2, Fig. 3) [163]. Preclinical studies in vitro and in vivo (mouse, rat, non-human primates: NHP) demonstrated no toxicity at cumulative doses of 100 mg/kg and stronger anti-tumor effects than standard chemotherapies in murine models [164, 165]. These promising results led to its first-in-human study in 1999 for the treatment of ALL and NHL. Apart from presenting a favorable pharmacokinetic profile in humans, no toxicity and a promising anti-tumor activity were reported [166, 167]. Unfortunately, the status of this compound has not been further reported (NCT00004858). Two additional studies investigated the antitumor activity of genistein conjugated to either anti-EGFR or 17.1A mAb, which targets an epithelial membrane antigen (Table 3). The anti-EGFR-genistein ADC demonstrated good tolerability up to 140 mg/kg, and significant anti-tumor activity at 1 mg/kg in preclinical models. 17.1A-genistein was found to be more active than unconjugated genistein in colon cancer models [168, 169].

More recently three other kinase inhibitors have been evaluated as ADC payloads. These molecules include neolymphostin (a PIKK inhibitor), and dasatinib and staurosporine, two multi-kinase inhibitors (Table 3, Fig. 4). Trastuzumab neolymphostin demonstrated selectivity and in vitro cytotoxicity despite being less potent than other usual trastuzumab-based ADCs [170]. An anti CXCR4 mAb coupled to dasatinib selectively delivered dasatinib to targeted T-cells and presented a strong immunosuppressive effect [171]. Lastly, the widely used laboratory reagent and multi-kinase inhibitor staurosporine was conjugated to cetuximab for the treatment of KRAS/BRAS mutated colon cancer cells [172]. Overall, the efficacy of tyrosine kinase inhibitors in ADC format is found to be limited and this family may not succeed in more advanced settings.

### Immune-stimulating antibody conjugates

Immune-stimulating antibody conjugates represent a new category of antibody–drug conjugates, with 2 ADCs currently in clinical trials (Table 2, Fig. 3) (NJH395, BDC-1001), and one, SBT6050, whose clinical evaluation has been terminated due to the sponsor’s strategic decision (NCT05091528). STING agonists and TLR agonist constitute the two main categories of conjugated immune stimulants.

The success of immune checkpoint inhibitors which target the adaptive immune system has greatly enhanced efforts to harness the stimulation of the innate immune system. However the systemic administration of the most potent agents such as STING and TLR agonists is associated with severe systemic toxicity, caused by a cytokine release syndrome, thereby restricting current studies to intra-tumor injections [173, 174]. Their conjugation to proteins or mAbs thus appears to be a promising means to exploit their strong antitumor potential while improving the tolerance profile.

Several immune-stimulating ADCs containing TLR agonists are currently being evaluated in the clinical setting. NJH395, which combines a small molecule TLR7/8 agonist with an anti-HER2 mAb is the first to have reached clinical evaluation (Table 2, Fig. 3). A phase I clinical trial in 18 patients with non-breast HER2 + malignancies (NCT03696771) showed severe toxicity including cytokine release syndrome, and lymphocyte depletion, in the absence of significant antitumor activity [175]. Similarly, BDC-1001, an immune-stimulating conjugate comprising an anti-HER2 antibody conjugated to a TLR7/8 agonist is currently under phase I/II evaluation for the treatment of patients with solid HER2 + tumors as a single agent or in combination with nivolumab (NCT04278144, Table 2, Fig. 3). Its preclinical evaluation demonstrated potent and durable immune-mediated antitumor efficacy and the clinical evaluation shows promising outcomes including no toxicity at the tested dose and evidence of clinical activity [176–178]. The analogue BDC-2034, targeting CEACAM5, has shown anti-tumor activity in vivo at low dose (0.5 mg/kg), activation of the innate immune system and reprogramming of intra-tumor myeloid, thus supporting its clinical development (Table 3) [179, 180]. The above-mentioned TLR7/8 analogue was also conjugated to an anti-PD-L1 antibody, aiming to combine immune checkpoint inhibition, antibody-dependent cellular phagocytosis (ADCP) and intra-tumor myeloid reprogramming [181]. This immune-stimulating ADC outperformed anti-PD-L1 anti-tumoral activity in preclinical models. SBT6050 is a pertuzumab-TLR8 agonist conjugate which is currently being evaluated as a single agent as well as in combination with anti PD1 inhibitors (NCT04460456) and with trastuzumab deruxtecan for the treatment of HER2-positive solid cancers (NCT05091528). Pertuzumab does not bind to the same HER2 epitope as trastuzumab and studies have demonstrated a synergistic potential between trastuzumab and SBT6050 [182, 183].

Promising preclinical results have been reported for other immune-stimulating ADCs, conjugated to either UC-1V150, CL264 or T785 TLR 7/8 agonists (Table 3, Fig. 4) [184, 185]. An anti-PD-L1 conjugated to the TLR7/8 agonist D18 has very recently been disclosed with promising preliminary results, including a potent anti-tumor activity in the B16 melanoma model which is a PD1-resistant model (Table 3, Fig. 4) [186]. Recently, a more selective agonist, i.e., TLR7 agonist has been investigated as immune-stimulating ADC payload [187]. The other emerging family of immune stimulant payloads are STING agonists. TAK-500 constitute the first STING agonist immune activating ADC to enter clinical trials and is currently recruiting patients (NCT05070247) [188]. This CCR2 directed ADC (TAK-676) is being evaluated for the treatment of solid tumors (Table 2, Fig. 3). In addition, three STING-conjugated ADCs are being developed at the preclinical stage: CDR-550, XMT-2056 and more recently a FcγR-targeting immune-stimulating ADC conjugated to the STING agonist XMT-1621 [189–192] (Table 3). The most advanced, XMT-2056 (STING agonist: XMT-1621, Fig. 4) leds to complete remissions of tumors at 1 mg/kg in mouse xenografts and demonstrated a synergistic activity with ICI while being tolerated in NHP with no clinical signs nor adverse histopathological findings. This promising ADC should enter clinical trials for a first-in-human study in 2022.

## Unconventional payloads at the preclinical stage

Since tumor cells have increased anabolic activity, several payload candidates have targeted various steps of protein synthesis, including transcription, splicing and translation inhibitors as well as protein catabolism. Another approach would be to target other ubiquitous cellular processes which are excessively active in neoplastic cells. However, only carefully designed ADCs may support this category of payload since even though the concentration of ADC is higher in tumors than in surrounding tissues, most of the compound administered intravenously does not localize to the tumor [193].

**Table 3:** Landscape of unconventional ADC payloads investigated at the preclinical stage

Payload mechanism	Payload family	Payload name {ADC name}	Target antigen	DAR	First publication date	Reference(s)
Topoisomerase II inhibitors	Anthracyclines	Doxorubicin	N/A	N/A	2010	109
			HER2	4	2017	110
			CD30	4	2017	110
		PNU-159682	LGR5	2	2015	111
			CD22	2	2015	114
			CD30	4	2017	109
			tenascin-C	2	2017	116
			HER2	4	2019	115
			CD46	2	2020	117
			EREG	n.d	2022	118
		PNU-159682 + MMAF	HER2	2 + 2	2019	119
		Daunorubicin	alpha-fetoprotein	1.2	1984	120
			ganglioside	20	1991	121
		Idarubicin	Ly2.1	2 to 4	1988	122
			CD19	3.2	1993	123
			HER2	1	2019	124
Transcription inhibitors	RNApolII inhibitors	Beta-amanitin	Albumin	1.9	1973	130
			MUC1	n. d	2006	131
			PSMA	2	2014	132, 133
		Alpha-amanitin and derivatives	Thy 1.2	3.6–6.3	1981	134
			EpCam	4 to 8	2012	137
			HER2	2	2018	138
			PSMA	2	2018	138
			CD19	2	2018	138
		Amanitin + MMAE	FGFR1	1 + 1	2018	139
		Phalloidin	Albumin	n. d	1986	140
		Trichothecene T-2	murine EL-4 lymphoma	n.d	1991	141
		Verrucarin A	oncofetal glycoprotein	n.d	1991	141
		Roridin A	oncofetal glycoprotein	n.d	1991	141
	Transcription Factor inhibitors	Triptolide	CD26	6.5	2019	145
			EGFR	5.5	2020	146
			HER2	2 to 3	2021	147
	HDAC inhibitors	ST7464AA1	EGFR	4.5	2018	150
			HER2	5	2020	151
		Vorinostat (SAHA)	EGFR, HER2	4 to 6	2021	152
		Dacinostat (VP-LAQ824)	EGFR, HER2	3	2021	152
Kinase inhibitors	PTK inhibitors	Genistein	EGFR	n.d	1998	168
			17.1A mAb	3	2003	169
	PIKK inhibitors	Neolymphostin	HER2	2	2019	170
	multi-kinase inhibitors	Dasatinib	CXCR4	3	2015	171
		Staurosporine	EGFR	n.d	2018	172
Immune stimulants	TLR7/8 agonists	UC-1V150	CD20	1 to 3	2015	184
		CL264	HER2	2	2020	185
		D18	PDL1	2	2021	186
		n.d	PDL1	2	2022	181
		n.d {BDC-2034}	CEACAM5	2.5	2021	179, 180
		T785	HER2	2	2020	185
	TLR7 agonists	AmberX ADC	HER2	n.d	2022	187
	STING agonists	{CRD5500}	HER2	n.d	2019	189
		XMT-1621	FcyR	2	2022	192
		XMT-1621 {XMT-2056}	HER2	8	2021	190, 191
Protein synthesis inhibitors	HSP90 inhibitors	Geldanamycin	HER2	n.d	2000	195, 196, 197
			CD70	4	2009	198
			HER2	n.d	2021	199
	Splicing inhibitors	Thailanstatin A	HER2	2 to 3	2016	25, 202
	Translation inhibitors	Psymberin (irciniastatin A)	CD30, CD70	5.4	2010	204
	Proteasome inhibitors	carmaphycin B analogues	HER2	1 to 2	2019	205
PROTACs/glue degraders	BET/BRD4 degraders	GNE-987	CCL1/HER2	6	2019	209
		MZ1 analogue	HER2	4	2020	212
			STEAP1	2 or 6	2021	210, 211
		BRD4/VHL	STEAP1/CCL1	6	2021	213
		BRD4/CRBN	HER2	2	2019	213
	ERa degraders	ERa/XIAP	HER2/CD22/B7-H4	2 to 6	2020	214
		ERa/VHL	HER2/CD22/B7-H4	2 to 6	2020	214
	TGFbR2 degraders	TGFbR2/VHL	HER2	2 to 4	2020	208
	BRM degraders	BRL/VHL	CD22	6	2020	208
	GSTP1 degraders	Smol006 {ORM-5029}	HER2	4	2022	215
Others	NAMPT inhibitors	FK-866 analogues	c-kit or HER2	2 to 4	2018	217
			CD30	8 to 10	2018	216
	KSP inhibitors	Filanesib derivative	HER2, c-kit	3 to 4.5	2019	218
			HER‐2, TWEAKR/Fn14	2 to 4	2018	219, 202
	carbonic anhydrase inhibitors	CA IX and XII peptides	CA IX and XII	n.d	2022	230
Beyond cytotoxic payloads	lipid homeostasis and inflammation	LXR agonist	CD11a	2	2016	235
	Enzymatic activity inhibition	CGS27023A	MMP9	4 to 8	2019	229

*ADC* Antibody–drug conjugate, *DAR* drug-to-antibody ratio

### HSP90 inhibitors

HSP90 (heat-shock protein 90) is a major chaperone protein which has been shown to be abnormally expressed in a variety of tumors. Several HSP90 inhibitors, derived from the geldanamycin (GA, Fig. 4) backbone, have been developed and tested in clinical settings. Upon binding to HSP90, inhibitors prevent its ability to protect its client proteins from proteasomal degradation [194]. Major limitations identified to date are significant dose-limiting toxicities and poor pharmacokinetic profiles. In the early 2000s, efforts have been made to chemically modify GA to synthesize a maleimide cleavable drug-linker suitable for bioconjugation (Table 3) [195–197]. The resulting trastuzumab-GA ADC demonstrated an increase of overall tumor-bearing mice survival compared to mice treated with trastuzumab. Streptonigrin and 17-amino-geldanamycin were used to generate anti-CD70 and anti-CD30 cleavable ADCs at a DAR4 and were found to be active in preclinical models (Table 3) [198]. A recent resurgence of GA in the payload landscape repositioned this molecule, by generating a HER2 scFv HBD/GA ADC that demonstrated anti-tumoral activity in a HER2-positive lung preclinical model (Table 3) [199].

### Splicing inhibitors

After transcription, pre-mRNA undergoes processing into mature mRNA by the removal of introns, achieved by the spliceosome. snRNPs (small nuclear ribonucleoproteins) U1, 2 4, 5 and 6 constitute the major snRNPs of the spliceosome. These complexes are essential to the generation of mature mRNA and are commonly deregulated in cancer cells. Targeting the SF3B1 subunit of U2 has been shown to efficiently inhibit splicing [200]. Several agents have been shown to be potent splicing inhibitors, including pladienolides, spliceostatins and thailanstatins. However, these highly cytotoxic molecules with IC_50_s in the nanomolar range were not further developed due to chemical instability. E7107, a pladienolide analogue, was evaluated in clinical trials (NCT00499499), but discontinued due to safety concerns, in particular severe ocular toxicity [201]. Thailanstatin A-trastuzumab conjugates were shown to be highly active in preclinical models, with greater potency than T-DM1 in certain in vivo models (Table 3, Fig. 4) [25, 202].

### Translation inhibitors

The development of tolerable translation inhibitors has proven to be challenging given the universal importance of translation in healthy tissues. Omacetaxine (previously designated homoharringtonine) is the first FDA-approved translation inhibitor and interferes with the initial elongation step of protein synthesis [203]. Several other translation inhibitors have been developed for the treatment of various cancers, targeting ribosomes, EIFs (eukaryotes translation initiation factors) or mTOR. To date only psymberin has been used as a potential ADC payload (Table 3, Fig. 4). Psymberin, also known as irciniastatin A, is a natural carbohydrate isolated from a marine sponge. Its conjugation to anti-CD30 and anti-CD70 antibodies through a beta-glucuronide linker demonstrated selectivity and anti-proliferative activity in vitro with IC_50_s in the sub-nanomolar range [204].

### Proteasome inhibitors

Proteasome inhibitors are an extremely potent class of anticancer agents. Bortezomib was approved in 2003 for the treatment of patients with multiple myeloma and has since then significantly improved the outcome of patients. Several other inhibitors have been developed, with reduced neurotoxic effects and/or allowing oral administration. Epoxyketone derivatives such as carmaphycin B analogues which strongly inhibit the 20S proteasome, have been conjugated to trastuzumab (Table 3, Fig. 4) [205]. Despite satisfactory in vitro cytotoxicity of the unconjugated payload, the corresponding ADC proved to be less potent than the corresponding MMAE-based ADC.

### PROTACS

Proteolysis Targeting Chimeric Molecules (PROTACs) are bifunctional molecules that bring together the E3 ligase with the target protein thus allowing its ubiquitination and degradation by the proteasome [206, 207]. Instead of directly inhibiting its target protein, PROTACs trigger its degradation with several potential clinical advantages such as prolonged effect, catalytic activity and therefore very potent cytotoxicity. Degrader-antibody conjugates (DACs) constitute an exciting emerging family in the ADC landscape. In DAC design, PROTACs can benefit from being transported by the mAb inside the cell to overcome their limited cell permeability. Current DACs constructions, biological activities and challenges have been reported in a recent review [208]. The BRD4/BET degraders, GNE-987, was conjugated to an anti-CLL1 antibody leading to a restored pharmacokinetic profile and potent in vivo activity in mice xenografts (Table 3, Fig. 4) [209]. MZI analogues conjugated to trastuzumab or an anti-STEAP1 antibody were also evaluated in vitro, demonstrating selective BRD4 degradation and cell cytotoxicity (Table 3, Fig. 4) [210–212]. Other BRD4 degrader-antibody conjugates, either comprising VHL or CRBN ligands have also recently been generated [213] (Table 3, Fig. 4). Similarly, Estrogen Receptor (ER), TGFbR2 and BRM degraders, are being investigated as DAC payloads by being conjugated to anti-HER2, anti-B7-H4 and/or anti-CD22 antibodies (Table 3, Fig. 4) [208, 214]. ORM-5029, the latest disclosed DAC or Antibody neoDegrader Conjugate (AnDC™), aims to deliver a GSPT1 degrader (Smol006) to HER2-expressing cells via pertuzumab. This AnDC™ has demonstrated stronger cytotoxicity than other GSPT1 degraders and anti-tumor activity comparable to that of DS-8201a [215]. The toxicity of ORM-5029 is currently under investigation and results will constitute the first report regarding the therapeutic window of DACs.

### Other molecules

Efforts in payload diversification have led to the recent preclinical development of unconventional antibody–drug conjugates delivering payloads with unique mechanism of action. Alteration of cellular metabolism by targeting nicotinamide phosphoribosyltransferase inhibitor (NAMPTs) constitutes a novel and original ADC technology. FK-866 analogues were conjugated to an anti-CD30 antibody and the subsequent ADC selectively depleted NAD in vitro and in vivo [216] (Table3, Fig. 4). CD30-NAMPTi demonstrated promising in vivo anti-tumor activity in xenografts with complete remissions at 3 mg/kg in the L540cy model. A favorable therapeutic index was outlined with a MTD greater than 100 mg/kg in rat. Other NAMPTis were also synthesized and conjugated to a c-Kit targeting mAb [217]. Despite selective and potent cytotoxicity in vitro (sub-nanomolar IC_50_s), these non-cleavable ADCs were moderately active in vivo with only partial responses at 20 mg/kg.

KSP (kinesin spindle protein) inhibitors, also named Eg5 inhibitors, constitute an emerging family of ADC payloads. Eg5 is a promising target for antitumor therapy since its expression is specific to proliferating cells and it is not expressed in cells of the nervous system. These payloads should therefore not present the neurological side effects classically associated with microtubule targeting agents. KSP inhibition prevents centrosome separation during cell division thus leading to mitotic arrest [218]. KSP inhibitor derivatives, with sub-nanomolar potency, were conjugated to HER2 and TWEAKR/Fn14 targeting antibodies (Table 3, Fig. 4) [219, 220]. The TWEAKR-KSPi ADC allowed a complete remission in a urothelial PDX while ispinesib, a small molecule KSP inhibitor, only delayed tumor growth in this model. ADCs have also been produced by conjugation of filanesib with an acceptable PK profile and good in vivo potency [221].

## Conclusions

**Fig. 5:**
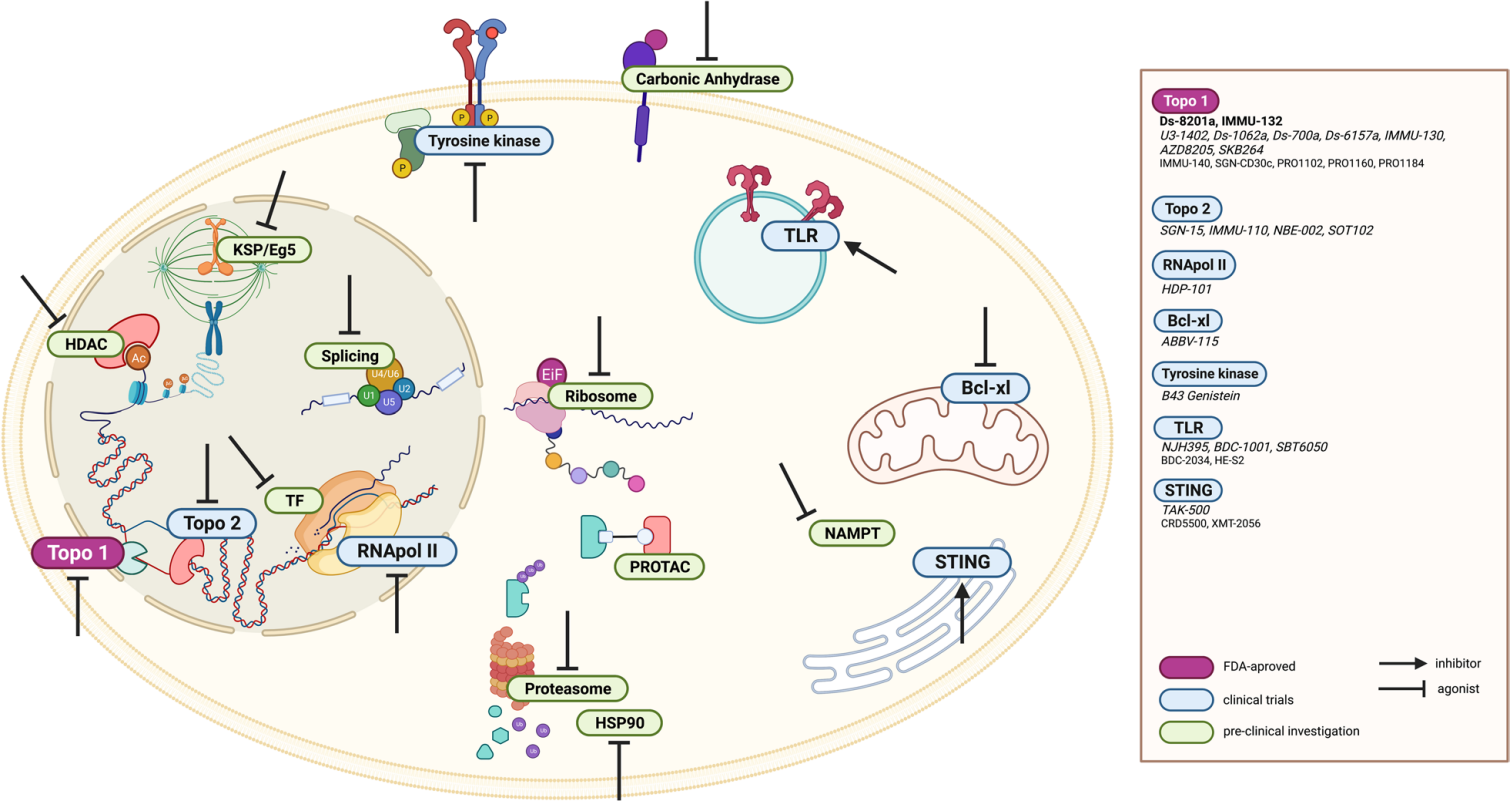
Schematic representation of the ADC payload’s target landscape beyond microtubules and DNA-intercalating agents. Notations: FDA-approved ADCs, *ADCs in clinical trials*

Antibody–drug conjugates have become an important component in the treatment of a growing number of cancer indications, and several hundred clinical trials are ongoing to explore novel targets and indications. The spectacular progress achieved in the ADC field is mainly supported by the tailoring of their design to a particular target. This has been rendered feasible thanks to several achievements including (1) the exploration and validation of a growing number of targets [222], (2) proper screening of mAbs specifically for ADC design, with a focus on cross-reactivities, preferential tumor binding helped by pH variations, decreased affinity to low nanomolar range to avoid stickiness and facilitate internalization and FcRn recycling, (3) improvements in conjugation technology enabling a higher drug-to-antibody ratio, and/or the restoration of the naked mAb-like pharmacokinetic profile [223] and (4) diversification of payloads (pictured in Fig. 5), as recently exemplified by the breakthroughs achieved with topoisomerase 1 inhibitors.

Among future developments, the continued diversification of payloads with original mechanisms of action is expected to play a key role. In advanced disease, cure is most generally achieved by combining agents with complementary mechanisms of action and, whenever possible, non-redundant toxicities. While the currently approved ADCs possess mechanisms of action which are similar to those of conventional chemotherapeutic agents, it is possible that future payloads will target vital cellular phenomena which have until now been intractable due to excessive toxicity. As previously observed with auristatins and maytansinoids conjugated with cleavable linkers, the bystander effect of topo-1 ADCs has demonstrated its effectiveness for the treatment of low or heterogeneous tumors and should be of growing interest in later development. Another major advantage of these novel payloads may rely on their ability to target quiescent tumor cells, which constitute the bulk of the tumor reservoir in patients. In addition, among the emerging payload families, several kinase inhibitors currently associated with severe side effects would benefit from a larger therapeutic index. For their part, PROTACs benefit from a substoichiometric activity that would in theory reduce the payload threshold for cytotoxicity.

Payload diversification also promises an opening of the ADC therapeutic arsenal to other cancers that do not yet benefit from targeted therapy. Sacituzumab govitecan led to the validation of TROP2 as a target in TNBC, and SN-38-based ADCs in clinical evaluation highlight the potential of new targets in cancer types that were poorly represented in the ADC landscape (HER3, CEACAM5, B7-H3 and GPR20). Interestingly, clinical trials against TNBC exhibit a growing number of antibody–drug conjugates comprising original payloads, including Dxd, PNU-159682 and SN-38 [224].

As we have aimed to describe in this review, several potential payloads have been identified and many have shown promising preclinical results. Some of these compounds have entered clinical trials but have not been pursued because of an unsatisfactory toxicity profile. In this regard, it should be emphasized that major technological advances, in particular the possibility to safely obtain ADCs with high drug-to-antibody ratios, support the fact that many of these payloads which were explored at a time when ADC production and characterization were suboptimal should be reconsidered with currently available technologies.

New ADC formats that integrate original payloads, such as dual payloads (Table 3), theranostic [225] and non-internalizing [226] conjugates have shown great potential in recent preclinical studies and may constitute growing fields in ADC research. Non-internalizing ADCs would particularly benefit from newer payloads that present a strong bystander killing effect [227] or that are directed against extracellular or stromal targets [228], as exemplified by the PNU-159682-based ADC targeting tenascin-C, the inhibition of matrix metalloproteinase extracellular protein or more recently the inhibition of carbonic anhydrases [116, 229, 230] (Table 3). Interestingly ADC technology is also being explored in non-oncological indications [231, 232]. Two original ADCs (ABBV-3373 and ABBV-154) containing a glucocorticoid receptor modulator (GRM) are being clinically evaluated for the treatment of rheumatoid arthritis and Crohn’s disease (Table 2, NCT03823391, NCT04888585, NCT05068284 and NCT04972968). Other immunology ADC payloads are being investigated in preclinical settings and could constitute an emerging class in ADC design [233, 234]. In addition, an A-rifamycin derivative, that demonstrated promising results in preclinical evaluation [235], has been investigated in phase 1 clinical trials in patients with *Staphylococcus aureus* bacteremia (NCT03162250). Non-cytotoxic payloads are also entering the payload landscape with the example of intracellular targeting of lipid metabolism by conjugating a Liver X Receptor (LXR) agonist to anti-CD11b antibody for the treatment of atherosclerosis (Table 3) [236].

Despite the broadened landscape of eligible diseases, a key issue for the development of these novel payloads will be to mitigate their side effects. Currently approved ADCs have shown that they are associated with expected (myelosuppression, neurotoxicity) or unexpected (such as ocular [237, 238] or pulmonary [68, 239]) toxicities. Obtaining a satisfactory therapeutic index will thus be an essential property for the future development of innovative ADC payloads.

## Data Availability

None. This manuscript is a review.
